# Co-existence of plasmid-mediated *bla*_NDM-1_ and *bla*_NDM-5_ in *Escherichia coli* sequence type 167 and ST101 and their discrimination through restriction digestion

**DOI:** 10.1128/spectrum.00987-24

**Published:** 2025-02-25

**Authors:** Amrita Bhattacharjee, Priyanka Basak, Shravani Mitra, Jagannath Sarkar, Shanta Dutta, Sulagna Basu

**Affiliations:** 1 Division of Bacteriology, ICMR-National Institute for Research in Bacterial Infections (Formerly ICMR-National Institute of Cholera and Enteric Diseases), Kolkata, West Bengal, India; 2 Department of Biological Sciences, Bose Institute, Kolkata, West Bengal, India; University of Pretoria, Pretoria, Gauteng, South Africa

**Keywords:** neonatal sepsis, epidemic clone, dual *bla*_NDM _variants, *bla*_NDM-1,-5_, nanopore, India

## Abstract

The concurrent presence of multiple New Delhi metallo-β-lactamase (*bla*_NDM_) variants within an isolate often goes undetected without next-generation sequencing. This study detects and characterizes dual *bla*_NDM_ variants in *Escherichia coli* through Sanger and whole-genome sequencing. Additionally, a rapid identification method utilizing restriction digestion was designed for detecting *bla*_NDM_ variants carrying M154L mutation. Antibiotic susceptibility, minimal inhibitory concentration for meropenem and ertapenem, PCR, and Sanger sequencing of *bla*_NDM_ along with genome sequencing using Illumina and Nanopore technology were conducted. Transmissibility and replicon types of *bla*_NDM_-harboring plasmids were evaluated. Restriction digestion using restriction enzyme, BtsCI was developed to distinguish between *bla*_NDM-1_ and *bla*_NDM_ variants possessing M154L mutation, such as *bla*_NDM-5_, *bla*_NDM-7_ etc. Two isolates belonging to phylogroups A; ST167 and B1; ST101 and resistant to meropenem and ertapenem (≥16 mg/L) were recovered from the blood of a neonate and the rectal swab of a pregnant woman, respectively. *bla*_NDM_ was detected by PCR, and Sanger sequences of *bla*_NDM_ showed two peaks at 262 (G and T) and 460 (A and C) nucleotide positions indicative of more than one *bla*_NDM_ variant. Hybrid assembly confirmed co-existence of *bla*_NDM-1_ and *bla*_NDM-5_ in each isolate. *bla*_NDM-1_ was located on IncY (ST167) and IncHI1A/HI1B (ST101), while *bla*_NDM-5_ was on IncFIA/FII (ST167) and IncC (ST101) plasmids in the two isolates. Digestion with BtsC1 could discriminate between *bla*_NDM-1_ and *bla*_NDM-5_. The co-existence of multiple *bla*_NDMs_, *bla*_NDM-1_, and *bla*_NDM-5_ in epidemic clones of *E. coli* is concerning. Restriction digestion method and Sanger sequencing can facilitate quick identification of dual *bla*_NDM_ variants in a single isolate.

**IMPORTANCE:**

The global dissemination of antimicrobial resistance genes is a serious concern. One such gene, *bla*_NDM_, has spread globally via plasmids. *bla*_NDM_ confers resistance against all β-lactam antibiotics, except monobactams. Most of the earlier literature reported the presence of single *bla*_NDM_ variant. However, this study reports the prevalence of dual *bla*_NDM_ variants (*bla*_NDM-1_ and *bla*_NDM-5_) located on two separate plasmids identified in two distinct *Escherichia coli* epidemic clones ST167 and ST101 isolated from a septicemic neonate and a pregnant mother, respectively. *bla*_NDM-5_ differs from *bla*_NDM-1_ due to the presence of two point mutations (i.e., V88L and M154L). This study detected dual *bla*_NDM_ variants through Sanger sequences and further validated them through hybrid-genome assembly. Detection of multiple *bla*_NDM_ variants in a single isolate remains difficult until genome sequencing or southern blotting is carried out. Hence, a simple restriction digestion method was devised to rapidly screen dual *bla*_NDM_ variants containing M154L mutation.

## OBSERVATION

New Delhi metallo-β-lactamase (*bla*_NDM_) is the fastest and most widespread carbapenemase that has triggered an alarming threat worldwide since its identification in 2009 (1, 2). Around 71 variants of *bla*_NDM_ have been reported worldwide, of which *bla*_NDM-1_, *bla*_NDM-5_, and *bla*_NDM-7_ are prevalent (3). NDM confers resistance against all β-lactam antibiotics, including carbapenems, the drug of last resort (4, 5).

Most studies report the presence of a single *bla*_NDM_ variant in a bacterial genome with few exceptions where two copies of *bla*_NDM-_variants (*bla*_NDM-1/NDM-5_) were present either in chromosome or plasmids in a single bacteria, such as *Pseudomonas aeruginosa*, *Escherichia coli*, *Klebsiella michiganensis*, and *Acinetobacter johnsonii* (Table S1) (6–12). *E. coli*, being a commensal and an opportunistic pathogen, acts as a reservoir of acquired antimicrobial resistance determinants eventually transferring it to other species (13).

In this study, we identified two *E. coli* isolates from blood (neonate) and rectal swab (adult) possessing two different variants of *bla*_NDM_. To the best of our knowledge, this is the first report where carriage of bi-variant *bla*_NDM_ (*bla*_NDM-1_ and *bla*_NDM-5_) in *E. coli* is being reported along with their characterization and genome analysis.

Two clinical *E. coli* isolates EN5349 and IN-MR210EC, were recovered from the blood of a septicemic neonate and the rectal swab of a hospitalized pregnant mother [as part of a collaborative study, called Burden of antibiotic resistance in neonates from developing societies (BARNARDS)], respectively. Isolates were assessed for antibiotic susceptibility by disk diffusion assay, and minimal inhibitory concentrations (MICs) for meropenem, ertapenem, and colistin (Sigma-Aldrich, Steinheim, Germany) were determined by the broth-micro dilution (14). PCR amplicons of *bla*_NDM_ were sequenced using primer pairs (Table S2) in an Applied Biosystems DNA analyzer (Perkin Elmer, USA) (15). Both short (Illumina NextSeq 500 platform, San Diego, CA) and long read-based (Oxford Nanopore, UK) sequencing technologies were used for genome sequencing. Unicycler was used to generate hybrid assemblies, which were further used for downstream analysis (Supplementary methods) (16). From hybrid assemblies, *bla*_NDM_ harboring plasmids were constructed and searched in the bacterial plasmid database (PLSDB) for similar complete plasmid sequences (17) . Plasmids showing nucleotide identity (≥99%) and same replicon types with similar *bla*_NDM_ variants with reference to study plasmids were compared.

Transfer of *bla*_NDM_ into *E. coli* J53 Az^r^ strain (100 mg/L) was attempted by solid-mating conjugation assay and electro-transformation into *E. coli* DH10B cells (Invitrogen, CA, USA) (Supplementary methods) (14, 15). Plasmid replicon types were determined by PCR-based replicon typing (PBRT, Diatheva, Italy) for wild type isolates (WTs) and transformants (TFs) (18, 19). PCR amplicons of *bla*_NDM_ gene were digested for 4 h using the BtsCI enzyme (New England Biolabs, USA) at 50°C.

Isolates belonged to phylogroups A; ST167 (EN5349) and B1; ST101 (IN-MR210EC), which are epidemic clones conferring resistance to carbapenems and other antibiotics while remaining susceptible to tigecycline and colistin (20). Presence of *bla*_NDM_ in isolates was confirmed by Sanger sequencing. Chromatogram of sequences (forward and reverse) for both isolates depicted the presence of two sharp peaks at 262 (G and T) and 460 (A and C) positions. Two different base calls at a single position suggested amplification of more than one *bla*_NDM_ in a single isolate (Fig. S1). G at position 262 and A at 460 corresponded to sequence of *bla*_NDM-1_, whereas G262→T (V88L) and A460→C (M154L) matched with *bla*_NDM-5_ (21). Meticulous evaluation of chromatogram helped the detection of two distinct *bla*_NDM_ copies.

Genome sequencing confirmed that isolates carried two copies of *bla*_NDM_ (*bla*_NDM-1_ and *bla*_NDM-5_), along with two copies of *bla*_TEM-1B_, which may increase enzyme production and enhance the chance of spread through various plasmids (15, 22). Both isolates EN5349 and IN-MR210EC exhibited a complex resistance profile, harboring multiple resistance determinants. These included genes conferring resistance to β-lactams (*bla*_NDM-1_, *bla*_NDM-5_, *bla*_TEM-1B_, *bla*_CTX-M-15_, *bla*_CMY-42_, *bla*_OXA-2_, *bla*_OXA-9_, *bla*_CMY-6_), aminoglycosides [*rmtB*, *rmtF*, *armA*, *aadA1*, *aadA2*, *aac(6')-Ib*, *aac(6')-Ib-cr*, *aph(6)-Id*, *aph(3'')-Ib*, *aac(3)-IIa*], sulfonamides (*sul1*, *sul2*), trimethoprim (*dfrA12*, *dfrA29*), phenicols (*catA1*), fluoroquinolones (*qnrS1*), and efflux pumps [*qacE*, *msr(E*), *mph(E*)] (Table 1). Additionally, IN-MR210EC carried multiple copies of *bla*_TEM-1A_, *sul1*, and *qacE* (Table 1). Presence of multiple resistance determinants indicated that several antibiotics would be ineffective against these isolates, implying a potential risk of treatment failure with the targeted antibiotics. The isolates exhibited susceptibility to tigecycline and colistin. However, the clinical utility of these two antibiotics is often limited due to potential adverse effects, such as nephrotoxicity with colistin and gastrointestinal disturbances with tigecycline (23, 24).

**TABLE 1:** Genome-based characterization of EN5349 and IN-MR210EC along with characterization of transformants (TFs) in terms of resistance determinants and plasmids^*a*^

Isolate/Accession number	EN5349/JAPTGK000000000	IN-MR210/JAYKKU000000000
Date of isolation	May, 2017	August, 2017
Source	Blood	Rectal swab
Phylogroup/Sequence types	A/ST167	B1/ST101
Virulence determinants		
Adherence	*ecpABCDER, elfACDG, eaeH, etpA, hcpABC, fimDFG, pilW*	*cfaABCDE, ecpABCDER, elfACDG, eaeH, hcpABC, fimABCDEFGHI, flgC, mrkABD*
Autotransporter	*cah, ehaB*	*ehaAB, upaG/ehaG*
Invasion	*ibeBC, tia*	*ibeBC, tia*
Iron uptake	*sitAD, fyuA, irp1, irp2, ybtAEPQSTUX, hemB*	*fyuA, irp1, irp2, ybtAEPQSTUX*
Toxin	*hlyE/clyA*	*hlyE/clyA*
Serum resistance	*iss, traT*	*iss*
Others	*Wzi, stjC, gale, mntB*	*lpfBCE*
Serotype/C-H type	O101:H9/11–0	O131:H31/41–191
Antibiotic susceptibility	Piperacillin^R^, Cefoxitin^R^, Cefotaxime^R^, Ciprofloxacin^R^, Trimethoprim-sulfamethoxazole^R^, Aztreonam^R^, Amikacin^R^, Gentamicin^R^, Meropenem^R^, Colistin^S^,Tigecycline^S^	Piperacillin^R^, Cefoxitin^R^, Cefotaxime^R^, Ciprofloxacin^R^, Trimethoprim-sulfamethoxazole^R^, Aztreonam^R^, Amikacin^R^, Gentamicin^R^, Meropenem^R^, Colistin^S^, Tigecycline^S^
Minimum inhibitory concentration (MIC)		
Meropenem	128 mg/L	16 mg/L
Ertapenem	64 mg/L	64 mg/L
Size of genome	5253 kb	5417 kb
	Chromosome 5014 kb	Chromosome 4820 kb
	Extra-chromosomal element 315 kb	Extra-chromosomal element 601 kb
GC content (%)	50.80%	50.40%
Core genome sequence type (cgST)	cgST169598	cgST28992
Resistance determinants		
(detected through PCR and WGS)		
Wild type	*bla*_*NDM-1*_ *, bla*_*NDM-5*_ *, bla*_*TEM-1B*_ *, bla*_*TEM-1B*_ *, bla*_*CTX-M-15*_ *, bla*_*CMY-42*_ *, rmtB, rmtF, aadA2, sul1, sul2, dfrA12, ARR-2, qacE, tet(A), mph(A), qnrS1, aac(6')-Ib-cr, aph(6)-Id, aph(3'')-Ib*	*bla*_NDM-1_*, bla*_NDM-5_*, bla*_TEM-1A_*, bla*_TEM-1A*,*_ *bla*_TEM-1B_*, bla*_TEM-1B_*, bla*_OXA-2_*, bla*_OXA-9_*, bla*_CMY-6_*, armA, rmtB, aadA1, aadA2, sul1, sul1, sul1, dfrA12, dfrA29, qacE, qacE, qacE, msr(E), mph(E), catA1, aac(6')-Ib, aac(6')-Ib-cr, aph(6)-Id, aph(3'')-Ib, aac(3)-IIa*
Resistance determinants (detected through PCR)		
TF1 (*bla*_NDM-1_^+ve^)	*bla*_*NDM-1*_ *,bla*_*TEM*_ *, bla*_*CTX-M*_ *,bla*_*CMY*_ *, qnrS, aac-(6’)-Ib-cr*	*bla*_*NDM-1*_ *,bla*_*TEM*_ *, aac(6')-Ib*
TF2 (*bla*_NDM-5_^+ve^)	*bla*_*NDM-5*_ *, bla*_*TEM*_ *, bla*_*CMY*_ *, rmtB*	*bla*_*TEM*_ *,rmtB*
TF3 (*bla*_NDM-1_^+ve^, *bla*_NDM-5_^+ve^)	*bla*_*NDM-1*_ *,bla*_*NDM-5*_ *, bla*_*TEM*_ *, bla*_*CTX-M*_ *, bla*_*CMY*_ *,rmtB, qnrS, aac-(6’)-Ib-cr*	NF
Plasmid replicon types (PBRT and WGS)		
Wild type	ColRNAI, IncFIA, IncFII, IncIγ, IncY	IncC, IncHI1A, IncHI1B
TF1 (*bla*_NDM-1_^+ve^)	IncY	IncC
TF2 (*bla*_NDM-5_^+ve^)	IncFII	IncHI1A, IncHI1B
TF3 (*bla*_NDM-1_^+ve^, *bla*_NDM-5_^+ve^)	IncFII, IncY	NF
MIC of Meropenem		
TF1 (*bla*_NDM-1_^+ve^)	64 mg/L	8 mg/L
TF2 (*bla*_NDM-5_^+ve^)	16 mg/L	8 mg/L
TF3 (*bla*_NDM-1_^+ve^, *bla*_NDM-5_^+ve^)	32 mg/L	NF
MIC of Ertapenem		
TF1 (*bla*_NDM-1_^+ve^)	64 mg/L	32 mg/L
TF2 (*bla*_NDM-5_^+ve^)	64 mg/L	32 mg/L
TF3 (*bla*_NDM-1_^+ve^, *bla*_NDM-5_^+ve^)	32 mg/L	NF
gyrA-parC mutations	*gyrA:p.S83L, gyrA:p.D87N, parC:p.S80I, parE:p.S458A*	*gyrA:p.S83L, gyrA:p.D87N, parC:p.S80I, parE:p.E460D*
Integrons	In27, In406	In27, In573
Characterization of *bla*_NDM-1_-carrying plasmid		
Plasmid Id	P1-EN5349	P1-IN-MR210EC
Size	122 kb	265 kb
Addiction system	relBE	relBE
Replicon type	IncY	IncHI1A, IncHI1B
Carriage of additional resistance determinants	*bla*_*TEM-1B*_ *, bla*_*CTXM-15*_ *, rmtF, sul2, ARR-2, qnrS1, aac-(6’)-Ib-cr, aph(6)-Id, aph(3”)Ib*	*bla*_TEM-1A_ *, bla*_OXA-9_ *, ∆bla*_DHA-1_ *, armA, aadA, sul1, qacE, msr(E), mph(E), aac(6')-Ib, aac(6')-Ib-cr*
Genetic Environment	IS*3000→*∆IS*Aba125*→ *bla*_NDM-1_→ *ble*_MBL_→ *trpF*→ *dsbD*→ *groES*→*groEL*→IS*3000*	IS*26*→ ∆IS*Aba125*→ *bla*_NDM-1_→ *ble*_MBL_→ ∆*bla*_DHA-1_ → *ampR*→ *sul1*→ IS*CR1*→ IS*5*
Characterization of *bla*_NDM-5_-carrying plasmid		
Plasmid Id	P2-EN5349	P2-IN-MR210EC
Size	125 kb	137 kb
Addiction system	*pemKI*	NF
Replicon type	FIA/FII (F36:A4:B-)	IncC
Carriage of additional resistance determinants	*bla*_*TEM-1B*_ *, rmtB, aadA2, sul1, dfrA12, qacE, tetA, mph(A)*	*bla*_TEM-1B_, *bla*_CMY-6_,*rmtB, aadA2, sul1, dfrA12, qacE*
Genetic Environment	IS*26*→∆IS*Aba125*→*bla*_NDM-5_ → *ble*_MBL_→ *trpF*→ *dsbD*→ IS*CR*→*sul1*→*aadA*→ *DuF1010*→ *dfrA12* → *IntI*→IS*26*	IS*26*→∆IS*Aba125*→*bla*_NDM-5_ → *ble*_MBL_ → *trpF* → *dsbD* → IS*CR1*→*sul1*→*aadA2*→*DuF1010*→*dfrA12*→*IntI1*→Tn*As2*

^*a*^ R, resistant; S, susceptible; TF, transformant; Inc, incompatibility; IS, insertion sequence; Int, integrase; NF, not found.

**Fig 1:**
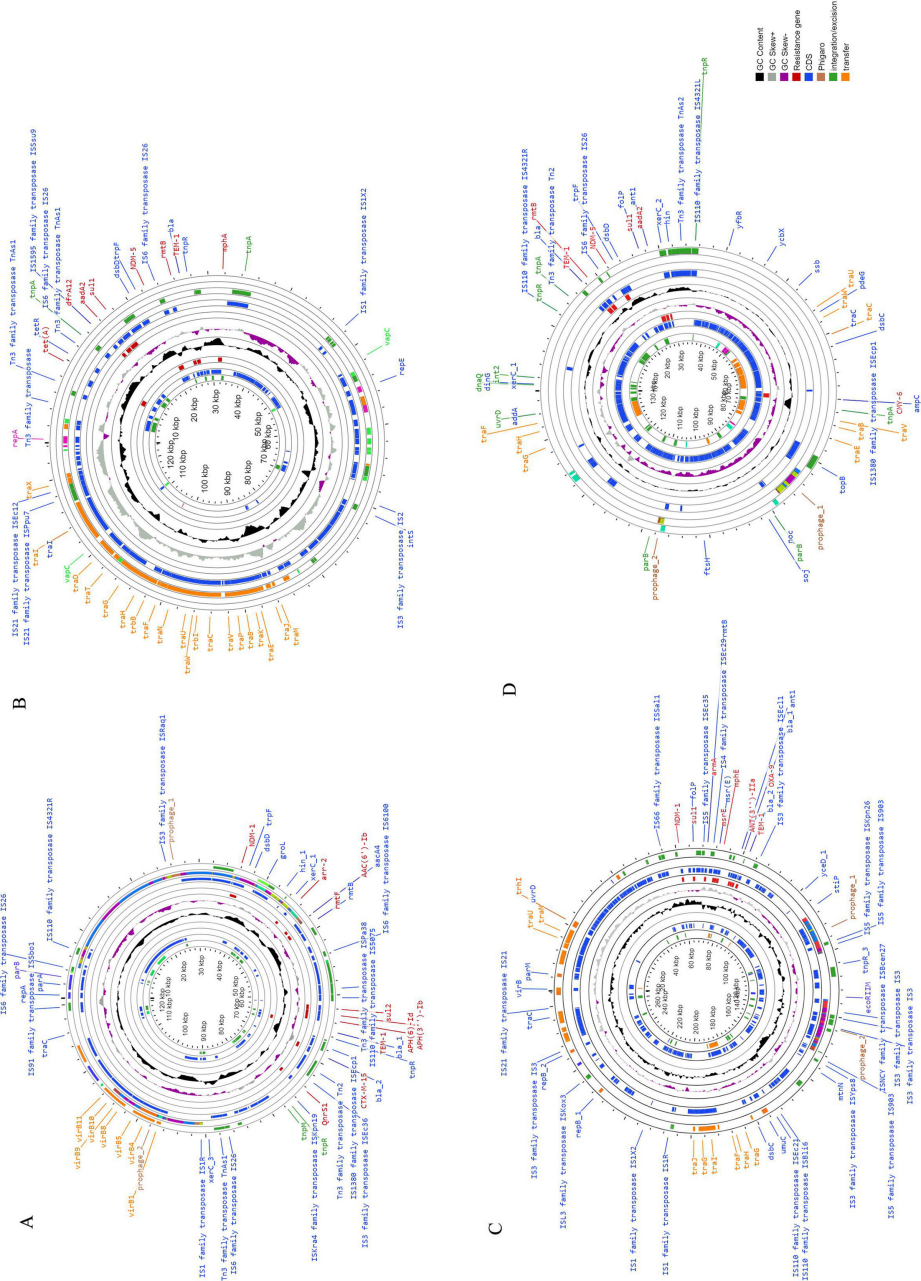
Circular map of the *bla*_NDM-1_ and *bla*_NDM-5_-harbouring plasmids in different *Escherichia coli,* EN5349 (ST167) and IN-MR210EC (ST101). Circular graphs representing the study plasmids, such as IncY [P1-EN5349; 122-kb] with *bla*_NDM-1_ (A), IncFIA/FII [P2-EN5349; 125-kb] with *bla_NDM-5_* (B)*,* IncHI1A/HI1B [P1-IN-MR210EC; 265-kb] with *bla*_NDM-1_ (C) and IncC [P2-IN-MR210EC; 137-kb] with *bla*_NDM-5_ (D). The inner circle is depicted as GC content in black and the outer circle represents GC skew in grey and purple. Resistance gene is denoted in red, coding sequences (CDS) in blue, prophage regions (phigaro) in brown, genes involved in transfer is in orange and integration/excision is in green.

Hybrid genome assembly revealed that *bla*_NDM-1_ and *bla*_NDM-5_ were harbored in two distinct plasmids, *viz. bla*_NDM-1_ in IncY [P1-EN5349; 122 kb] and IncHI1A/HI1B [P1-IN-MR210EC; 265 kb], whereas *bla*_NDM-5_ were in IncFIA/FII [P2-EN5349; 125 kb] and IncC [P2-IN-MR210EC; 137 kb] (Fig. 1). Earlier studies have reported the occurrence of double copies of same *bla*_NDM_ variant, either located in the chromosome or in plasmids, among different organisms (Table S1) (6–12). This study reports different *bla*_NDM_ variants in a single *E. coli* isolate.

The genetic environment of *bla*_NDM-1_ featured a truncated IS*Aba125* upstream, along with Tn*3*-like IS*3000* and IS*26*-like family transposase located further upstream of IS*Aba125* in EN5349 and IN-MR210EC, respectively. The downstream regions of *bla*_NDM-1_ varied (Table 1). Moreover, for *bla*_NDM-5_, both isolates possessed an IS*26*-like family transposase and ∆IS*Aba125* upstream, with *ble*_MBL_ downstream, followed by *trpF*, *dsbD*, and IS*91*-like IS*CR1* family transposase (Table 1). The genetic environment of *bla*_NDM-5_ in study plasmids, P2-EN5349 and P2-IN-MR210EC showed >95% similarity (Fig. S2). Genetic environments of the study *bla*_NDM_ variants were individually comparable with the genetic backgrounds of the respective *bla*_NDM_ variants reported worldwide (4, 15, 25).

Conjugation of study plasmids was unsuccessful, and transformants obtained by electroporation carried either *bla*_NDM-1_ or *bla*_NDM-5_. Few TFs of EN5349 (EN5349.TF3) co-harbored *bla*_NDM-1_ and *bla*_NDM-5_ as confirmed by Sanger sequencing. WT and TFs exhibited high MIC values for meropenem and ertapenem (≥16 mg/L) (Table 1). *bla*_NDM_ has been known to be promiscuous in nature, and it is easily transmitted between organisms (4, 26). Study plasmids, such as P2-EN5349, P1-IN-MR210EC, and P2-IN-MR210EC, possessed plasmid transfer/mobilization factors (*tra*-operon system, *mob*I), and P1-EN5349 possessed conjugation/type IV secretion system (T4SS) (*virB*, *virB9*, *virB11*, *virB6*, *virB4*, *Rhs*) (Fig. 1) (27, 28) analyzed through genome sequences. Although conjugation was unsuccessful under laboratory conditions, the presence of such genes in plasmids still suggests the potential for *bla*_NDM_ transmission in natural environments. Such transmission may occur not only in the hospital environment but also in other environments. Multiple *bla*_NDM_ variants in *E. coli* epidemic clones increase the potential for transmission, along with transfer of other resistance genes present in the same plasmid (14, 20).

**Fig 2:**
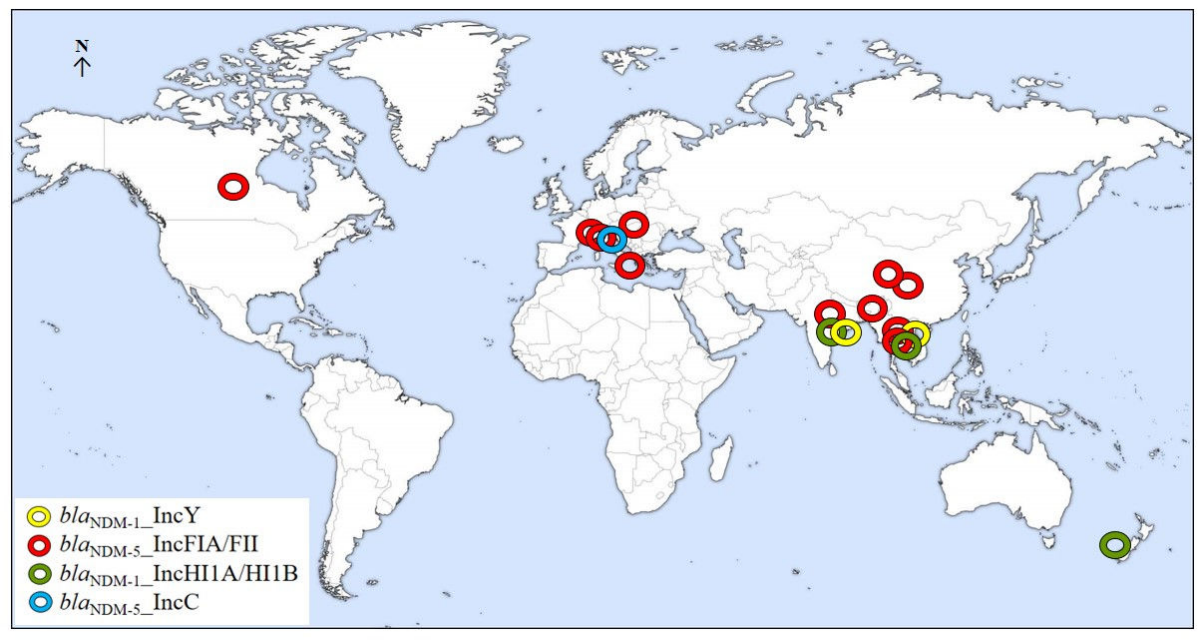
Worldwide prevalence of plasmids similar to study plasmids. Globally reported plasmids similar (≥99% nucleotide identity) to study plasmids are denoted in four different colours such as yellow (IncY with *bla*_NDM-1_), red (IncFIA/FII with *bla*_NDM-5_), green (IncHI1A/HI1B with *bla*_NDM-1_) and sky blue (IncC with *bla*_NDM-5_). The map was obtained from freeworldmaps.net.

Study plasmids showed close resemblance with some globally reported plasmids harboring similar *bla*_NDM_ variants and same replicon types in different species of Enterobacterales. P2-EN5349 (pMLST F36:A4:B-) showed similarity with 11 globally reported IncFIA/FII plasmids harboring the *bla*_NDM-5_ gene. These plasmids were identified in diverse *E. coli* isolates obtained from clinical specimens (rectal swabs, urine, faecal swabs, and mastitis milk) across various countries, including Canada, the Czech Republic, Switzerland, Italy, Myanmar, Bangladesh, China, Thailand, and India (Fig. 2; Table S3) (17). In contrast, P2-IN-MR210EC (*bla*_NDM-5_ in IncC plasmid) exhibited similarity only to a plasmid from environmental *E. coli* found in Switzerland. These findings suggest a more widespread dissemination of *bla*_NDM-5_ via IncFIA/FII plasmids compared to IncC. There were very few reports of *bla*_NDM-1_ transmission via IncY and IncHI1A/HI1B plasmids. Notably, P1-IN-MR210EC (*bla*_NDM-1_ in IncHI1A/HI1B) shared similarities with five plasmids found in clinical *K. pneumoniae* isolates from India, Thailand, and New Zealand. (Fig. 2; Table S3) (17).

With the emergence of organisms carrying two different variants of *bla*_NDM_ in different plasmids, spread of such variants and their stability in antibiotic-loaded environment calls for timely identification of such strains. Dual *bla*_NDM_ variants in a single isolate may be confirmed by southern blotting and hybrid assembly, which are expensive, time-consuming, and difficult to execute for a diagnostic laboratory. Hence, a novel restriction digestion-based method was introduced to distinguish between *bla*_NDM_ variants (NDM-4, 5, 7, 8, 12, 13, 15, 16b, 17, 19, 20, 21, 27, 35, 36, 37) with or without M154L mutation. *bla*_NDM-1_ has two recognition sites for BtsCI (Fig. S3), which generate three DNA fragments (255, 215, and 355 bp). Presence of a mutation (A460→C) corresponding to M154L alters the second recognition site (GGATG→GGCTG), which BtsCI is unable to cleave, resulting in two fragments of 255 and 570 bp (Fig. S3). Hence, most M154L possessing variants (including *bla*_NDM-5_) will produce fragments different from the *bla*_NDM-1_ variant. Since study isolates possessed both *bla*_NDM-1_ and *bla*_NDM-5_, four DNA fragments, that is, 215, 255, 355, and 570 bp (Fig. S3), were generated, which confirmed the presence of *bla*_NDM-1_ along with a variant possessing M154L mutation (4). Identification of resistance genes in diagnostic laboratories, especially in low- and middle-income countries (LMICs), still remains limited due to the lack of access to PCR-based molecular techniques. Diagnostic laboratories rely on conventional disk diffusion tests or MIC values generated through automated systems, which further guide their treatment decisions. While whole-genome sequencing (WGS) offers a comprehensive approach for identifying resistance determinants, its high cost and lack of expertise in data analysis limit its widespread adoption in LMIC settings.

Our study adds to the very few studies that reported more than one variant of *bla*_NDM_ in a single isolate. The presence of multiple *bla*_NDM_ variants in *E. coli* epidemic clones collected from a septicemic neonate and a pregnant mother (not paired) is worrisome. Emergence and spread of such organisms are of immense public health consequence. Two different variants of *bla*_NDM_ in different plasmids and their stability in antibiotic-loaded environment calls for timely identification.

## Data Availability

Genome data were submitted to the NCBI database with accession numbers JAPTGK000000000 and JAYKKU000000000 (Table 1).

## SUPPLEMENTAL MATERIAL

Supplemental material spectrum.00987-24-s0001.docx Supplemental methods, Tables S1 to S3, and Figs. S1 to S3.

ASM does not own the copyrights to Supplemental Material that may be linked to, or accessed through, an article. The authors have granted ASM a non-exclusive, world-wide license to publish the Supplemental Material files. Please contact the corresponding author directly for reuse.

## References

[1] Kumarasamy KK, Toleman MA, Walsh TR, Bagaria J, Butt F, Balakrishnan R, Chaudhary U, Doumith M, Giske CG, Irfan S, et al.. 2010. Emergence of a new antibiotic resistance mechanism in India, Pakistan, and the UK: a molecular, biological, and epidemiological study. Lancet Infect Dis 10:597–602. doi:10.1016/S1473-3099(10)70143-2 20705517 PMC2933358

[2] Yong D, Toleman MA, Giske CG, Cho HS, Sundman K, Lee K, Walsh TR. 2009. Characterization of a new metallo-β-lactamase gene, bla_NDM-1_, and a novel erythromycin esterase gene carried on a unique genetic structure in Klebsiella pneumoniae sequence type 14 from India. Antimicrob Agents Chemother 53:5046–5054. doi:10.1128/AAC.00774-09 19770275 PMC2786356

[3] Paul D, Babenko D, Toleman MA. 2020. Human carriage of cefotaxime-resistant Escherichia coli in North-East India: an analysis of STs and associated resistance mechanisms. J Antimicrob Chemother 75:72–76. doi:10.1093/jac/dkz416 31622465

[4] Khan AU, Maryam L, Zarrilli R. 2017. Structure, genetics and worldwide spread of New Delhi metallo-β-lactamase (NDM): a threat to public health. BMC Microbiol 17:101. doi:10.1186/s12866-017-1012-8 28449650 PMC5408368

[5] Papp-Wallace KM, Endimiani A, Taracila MA, Bonomo RA. 2011. Carbapenems: past, present, and future. Antimicrob Agents Chemother 55:4943–4960. doi:10.1128/AAC.00296-11 21859938 PMC3195018

[6] Jiang B, Ji X, Lyu Z-Q, Liang B, Li J-H, Zhu L-W, Guo X-J, Liu J, Sun Y, Liu Y-J. 2022. Detection of two copies of a bla_NDM-1_-encoding plasmid in Escherichia coli isolates from a pediatric patient with diarrhea. Infect Drug Resist 15:223–232. doi:10.2147/IDR.S346111 35115791 PMC8801394

[7] Shen P, Yi M, Fu Y, Ruan Z, Du X, Yu Y, Xie X. 2017. Detection of an Escherichia coli sequence type 167 strain with two tandem copies of bla_NDM-1_ in the chromosome. J Clin Microbiol 55:199–205. doi:10.1128/JCM.01581-16 27807154 PMC5228230

[8] Feng Y, Liu L, McNally A, Zong Z. 2018. Coexistence of two bla_NDM-5_ genes on an IncF plasmid as revealed by nanopore sequencing. Antimicrob Agents Chemother 62. doi:10.1128/AAC.00110-18 PMC5923099 29439976

[9] Jovcić B, Lepsanović Z, Begović J, Rakonjac B, Perovanović J, Topisirović L, Kojić M. 2013. The clinical isolate Pseudomonas aeruginosa MMA83 carries two copies of the bla_NDM-1_ gene in a novel genetic context. Antimicrob Agents Chemother 57:3405–3407. doi:10.1128/AAC.02312-12 23612199 PMC3697382

[10] Tang L, Shen W, Zhang Z, Zhang J, Wang G, Xiang L, She J, Hu X, Zou G, Zhu B, Zhou Y. 2020. Whole-genome analysis of two copies of bla_NDM-1_ gene carrying Acinetobacter johnsonii strain Acsw19 isolated from Sichuan, China. Infect Drug Resist 13:855–865. doi:10.2147/IDR.S236200 32273730 PMC7106997

[11] Zheng B, Xu H, Yu X, Lv T, Jiang X, Cheng H, Zhang J, Chen Y, Huang C, Xiao Y. 2018. Identification and genomic characterization of a KPC-2-, NDM-1- and NDM-5-producing Klebsiella michiganensis isolate. J Antimicrob Chemother 73:536–538. doi:10.1093/jac/dkx415 29126236

[12] Yang L, Lin Y, Lu L, Xue M, Ma H, Guo X, Wang K, Li P, Du X, Qi K, Li P, Song H. 2020. Coexistence of two bla_NDM–5_ genes carried on IncX3 and IncFII plasmids in an Escherichia coli isolate revealed by Illumina and Nanopore sequencing. Front Microbiol 16. doi:10.3389/fmicb.2020.00195 PMC7031209 32117184

[13] Basu S. 2020. Variants of the New Delhi metallo-β-lactamase: new kids on the block. Future Microbiol 15:465–467. doi:10.2217/fmb-2020-0035 32378966

[14] Bhattacharjee A, Sands K, Mitra S, Basu R, Saha B, Clermont O, Dutta S, Basu S. 2023. A decade-long evaluation of neonatal septicaemic Escherichia coli: clonal lineages, genomes, and New Delhi metallo-β-lactamase variants. Microbiol Spectr 11:e0521522. doi:10.1128/spectrum.05215-22 37367488 PMC10434172

[15] Datta S, Mitra S, Chattopadhyay P, Som T, Mukherjee S, Basu S. 2017. Spread and exchange of bla _NDM-1_ in hospitalized neonates: role of mobilizable genetic elements. Eur J Clin Microbiol Infect Dis 36:255–265. doi:10.1007/s10096-016-2794-6 27796645

[16] Wick RR, Judd LM, Gorrie CL, Holt KE. 2017. Unicycler: resolving bacterial genome assemblies from short and long sequencing reads. PLOS Comput Biol 13:e1005595. doi:10.1371/journal.pcbi.1005595 28594827 PMC5481147

[17] Schmartz GP, Hartung A, Hirsch P, Kern F, Fehlmann T, Müller R, Keller A. 2022. PLSDB: advancing a comprehensive database of bacterial plasmids. Nucleic Acids Res 50:D273–D278. doi:10.1093/nar/gkab1111 34850116 PMC8728149

[18] Carattoli A, Bertini A, Villa L, Falbo V, Hopkins KL, Threlfall EJ. 2005. Identification of plasmids by PCR-based replicon typing. J Microbiol Methods 63:219–228. doi:10.1016/j.mimet.2005.03.018 15935499

[19] Villa L, García-Fernández A, Fortini D, Carattoli A. 2010. Replicon sequence typing of IncF plasmids carrying virulence and resistance determinants. J Antimicrob Chemother 65:2518–2529. doi:10.1093/jac/dkq347 20935300

[20] Cummins EA, Snaith AE, McNally A, Hall RJ. 2021. The role of potentiating mutations in the evolution of pandemic Escherichia coli clones. Eur J Clin Microbiol Infect Dis. doi:10.1007/s10096-021-04359-3 34787747

[21] Nakano R, Nakano A, Hikosaka K, Kawakami S, Matsunaga N, Asahara M, Ishigaki S, Furukawa T, Suzuki M, Shibayama K, Ono Y. 2014. First report of metallo-β-lactamase NDM-5-producing Escherichia coli in Japan. Antimicrob Agents Chemother 58:7611–7612. doi:10.1128/AAC.04265-14 25246390 PMC4249512

[22] Maddamsetti R, Yao Y, Wang T, Gao J, Huang VT, Hamrick GS, Son H-I, You L. 2024. Duplicated antibiotic resistance genes reveal ongoing selection and horizontal gene transfer in bacteria. Nat Commun 15:1449. doi:10.1038/s41467-024-45638-9 38365845 PMC10873360

[23] Kilic I, Ayar Y, Ceylan İ, Kaya PK, Caliskan G. 2023. Nephrotoxicity caused by colistin use in ICU: a single centre experience. BMC Nephrol 24:302. doi:10.1186/s12882-023-03334-8 37833622 PMC10576281

[24] Greer ND. 2006. Tigecycline (Tygacil): the first in the glycylcycline class of antibiotics. Proc (Bayl Univ Med Cent) 19:155–161. doi:10.1080/08998280.2006.11928154 16609746 PMC1426172

[25] Mitra S, Mukherjee S, Naha S, Chattopadhyay P, Dutta S, Basu S. 2019. Evaluation of co-transfer of plasmid-mediated fluoroquinolone resistance genes and bla_NDM_ gene in Enterobacteriaceae causing neonatal septicaemia. Antimicrob Resist Infect Control 8:46. doi:10.1186/s13756-019-0477-7 30858970 PMC6391786

[26] Wu W, Feng Y, Tang G, Qiao F, McNally A, Zong Z. 2019. NDM metallo-β-lactamases and their bacterial producers in health care settings. Clin Microbiol Rev 32:e00115-18. doi:10.1128/CMR.00115-18 30700432 PMC6431124

[27] Virolle C, Goldlust K, Djermoun S, Bigot S, Lesterlin C. 2020. Plasmid transfer by conjugation in gram-negative bacteria: from the cellular to the community level. Genes (Basel) 11:1239. doi:10.3390/genes11111239 33105635 PMC7690428

[28] Christie PJ. 2016. The mosaic type IV secretion systems. EcoSal Plus 7. doi:10.1128/ecosalplus.ESP-0020-2015 PMC5119655 27735785

