# Supplementary material for: Co-existence of plasmid-mediated blaNDM-1 and blaNDM-5 in Escherichia coli sequence type 167 and ST101 and their discrimination through restriction digestion
Source: Microbiol Spectr. 2025 Feb 25;13(4):e00987-24. doi: 10.1128/spectrum.00987-24 (PMC11960126; doi:10.1128/spectrum.00987-24)
Supplement: Supplemental material — Supplemental methods, Tables S1 to S3, and Figs. S1 to S3. [file spectrum.00987-24-s0001.docx]

**Supplementary data**

**Supplementary Methods:**

1. **Whole genome sequencing (WGS)-**

Genomic DNA was extracted using a Wizard Genomic DNA Purification Kit (Promega). The library preparation was carried out using a Nextera XT Kit and Ligation sequencing kit for short- and long-read sequencing, respectively. Sequenced short- and long-reads were filtered out that having an average quality value less than 20 and 7, by using Prinseq (1) and Nanofilt (2), respectively. Unicycler (3) was used to generate the hybrid mode of assembly which uses short reads to generate contiguous sequences and long reads for gap-filling purpose only. Assembly files were annotated using Rapid annotation using subsystem technology (RAST) (4).

Assembly files were used for different downstream analyses. Resistance determinants, virulence determinants, Multilocus sequence typing (MLST), *fumC*-*fimH* (C-H) typing, serotyping, plasmid replicon typing, plasmid-multilocus sequence typing (pMLST) and mobile genetic elements (MGE) of the isolates were analysed using online free database (<http://www.genomicepidemiology.org/>). Genetic environment of *bla*_NDM_ was visualised in SnapGene viewer tool. Circular map of *bla*_NDM_-carrying plasmid was generated using the online tool Proksee-Genome Analysis (5).

1. **Transformation assay**

Electro-transformation was achieved using isolated plasmids (PureYield™ Plasmid Midiprep System, Promega, United States) and transformed to recipient *E. coli* DH10B cells (Invitrogen, CA, USA). Transformants (TFs) were selected on LB agar (Difco™, Lennox) supplemented with 2mg/L meropenem (Sigma-Aldrich, USA) and presence of *bla*_NDM_ was confirmed by PCR. MIC values of TFs were determined for meropenem and ertapenem (6).

**References**

1. Schmieder R, Edwards R. 2011. Quality control and preprocessing of metagenomic datasets. Bioinformatics 27:863–864.

2. De Coster W, D’Hert S, Schultz DT, Cruts M, Van Broeckhoven C. 2018. NanoPack: visualizing and processing long-read sequencing data. Bioinformatics 34:2666–2669.

3. Wick RR, Judd LM, Gorrie CL, Holt KE. 2017. Unicycler: Resolving bacterial genome assemblies from short and long sequencing reads. PLOS Comput Biol 13:e1005595.

4. Aziz RK, Bartels D, Best AA, DeJongh M, Disz T, Edwards RA, Formsma K, Gerdes S, Glass EM, Kubal M, Meyer F, Olsen GJ, Olson R, Osterman AL, Overbeek RA, McNeil LK, Paarmann D, Paczian T, Parrello B, Pusch GD, Reich C, Stevens R, Vassieva O, Vonstein V, Wilke A, Zagnitko O. 2008. The RAST Server: rapid annotations using subsystems technology. BMC Genomics 9:75.

5. Grant JR, Enns E, Marinier E, Mandal A, Herman EK, Chen C-Y, Graham M, Van Domselaar G, Stothard P. 2023. Proksee: in-depth characterization and visualization of bacterial genomes. Nucleic Acids Res 51:W484–W492.

6. Datta S, Mitra S, Chattopadhyay P, Som T, Mukherjee S, Basu S. 2017. Spread and exchange of bla NDM-1 in hospitalized neonates: role of mobilizable genetic elements. Eur J Clin Microbiol Infect Dis Off Publ Eur Soc Clin Microbiol 36:255–265.

**Table S1. Publications reporting the existence of two copies of *bla*_NDM_ in Gram-negative bacteria**

| **Year of Publication** | **Species origin** | **Reported *bla*_NDM_ variants** | **Country of origin** | **Sample Sources** | **Position of *bla*_NDM_in genome** | **DOI and PMID** |
| --- | --- | --- | --- | --- | --- | --- |
| 2022 | *Escherichia coli* | 2 copies of *bla*_NDM-1_ | China | faecal sample, 2019 | Single IncC (~192-kb) | DOI: [10.2147/IDR.S346111](https://doi.org/10.2147/idr.s346111)  PMID: 35115791 |
| 2020 | *Escherichia coli* | 2 copies of *bla*_NDM-5_ | Guangzhou, China | faecal sample, 2018 | IncX3 (~55-kb),  IncFII (~65-kb) [F2:A-:B-] | DOI: [10.3389/fmicb.2020.00195](https://doi.org/10.3389/fmicb.2020.00195) PMID: 32117184 |
| 2020 | *Acinetobacter johnsonii* | 2 copies of *bla*_NDM-1_ | Sichuan, China | municipal sludge, 2019 | Chromosome (~3.43-Mbp)  Plasmid (~352-kb) | DOI: [10.2147/IDR.S236200](https://doi.org/10.2147/idr.s236200)  PMID: 32273730 |
| 2018 | *Escherichia coli* | 2 copies of *bla*_NDM-5_ | China | urine sample, 2016 | Single IncFII (~145-kb) [F36:A4:B-] | DOI: [10.1128/AAC.00110-18](https://doi.org/10.1128%2FAAC.00110-18)  PMID: [29439976](https://pubmed.ncbi.nlm.nih.gov/29439976) |
| 2018 | *Klebsiella michiganensis* | *bla*_NDM-1_ and *bla*_NDM-5_ | Zhejiang, China | faecal sample, 2016 | IncF (~106-kb)  IncX3 (~46-kb) | DOI: [10.1093/jac/dkx415](https://doi.org/10.1093/jac/dkx415)  PMID: 29126236 |
| 2016 | *Escherichia coli* | 2 copies of *bla*_NDM-1_ | China | urine Sample, 2013 | Chromosome (~4.8-Mbp) | doi: [10.1128/JCM.01581-16](https://doi.org/10.1128%2FJCM.01581-16)  PMID: [27807154](https://pubmed.ncbi.nlm.nih.gov/27807154) |
| 2013 | *Pseudomonas aeruginosa* | 2 copies of *bla*_NDM-1_ | Serbia | Not mentioned | Chromosome | doi: [10.1128/AAC.02312-12](https://doi.org/10.1128%2FAAC.02312-12)  PMID: [23612199](https://pubmed.ncbi.nlm.nih.gov/23612199) |

**Table S2: Primers used for PCR, Sanger sequencing of *bla*_NDM_ and restriction digestion method**

| Primer | Primer Sequence (5’-3’) | Product size (bp) |
| --- | --- | --- |
| NDM_Forward | 5’-CATATGATGGAATTGCCCAATATTATG-3’ | 825 |
| NDM_Reverse | 5’-CTCGAGTCAGCGCAGCTTGTCGG-3’ |  |

**Table S3. List of plasmids with their NCBI accession numbers exhibiting homology with the study plasmids detected through the plasmid database (PLSDB)**

| **Plasmid symbol** | **Study plasmid Id** | **Accession number of global plasmids** | **Country of origin** | **NDM-variants** | **Reported organisms** | **Isolation Source** |
| --- | --- | --- | --- | --- | --- | --- |
|  | P1-EN5349 (NDM-1) | NZ_KF220658.1 | India | NDM-1 | *Klebsiella pneumoniae* | Clinical |
|  |  | NZ_MN370928.1 | Thailand | NDM-1 | *Klebsiella pneumoniae* | Human intestinal flora |
|  | P2-EN5349 (NDM-5) | NZ_CP048374.1 | Switzerland | NDM-5 | *Escherichia coli* | Environment |
|  |  | NZ_MH917716.1 | China | NDM-5 | *Escherichia coli* | - |
|  |  | NZ_MN007141.1 | Italy: Rome | NDM-5 | *Escherichia coli* | Fecal swab |
|  |  | NZ_CP050384.1 | Czech Republic, Ostrava | NDM-5 | *Escherichia coli* | Urine |
|  |  | NZ_CP033159.1 | India | NDM-5 | *Escherichia coli* | Mastitis Milk |
|  |  | NZ_MF156713.1 | Beijing, China | NDM-5 | *Escherichia coli* | - |
|  |  | NZ_AP018833.1 | Myanmar, Yangon | NDM-5 | *Escherichia coli* | Clinical |
|  |  | CP023871.1 | Canada | NDM-5 | *Escherichia coli* | Rectal swab |
|  |  | CP083702.1 | Switzerland: Basel | NDM-5 | *Escherichia coli* | Urine |
|  |  | CP095643.1 | Bangladesh: Dhaka | NDM-5 | *Escherichia coli* | Urine |
|  |  | [LC744462.1](https://www.ncbi.nlm.nih.gov/nucleotide/LC744462.1?report=genbank&log$=nucltop&blast_rank=11&RID=FTJ0H3XW013) | Myanmar | NDM-5 | *Escherichia coli* | - |
|  | P1-IN-MR210EC  (NDM-1) | NZ_CP028171.1 | India | NDM-1 | *Salmonella enterica* | Biological fluid-human |
|  |  | NZ_MN370929.1 | Thailand | NDM-1 | *Klebsiella pneumoniae* | Human intestinal flora |
|  |  | NZ_KF220658.1 | India | NDM-1 | *Klebsiella pneumoniae* | Bile fluid |
|  |  | NZ_MN370927.1 | Thailand | NDM-1 | *Klebsiella pneumoniae* | Human intestinal flora |
|  |  | NZ_CP049968.1 | New Zealand | NDM-1 | *Escherichia coli* | Urine |
|  | P2-IN-MR210EC (NDM-5) | NZ_CP048374.1 | Switzerland | NDM-5 | *Escherichia coli* | Environment |

The plasmid database (PLSDB), https://ccb-microbe.cs.uni-saarland.de/plsdb20

**
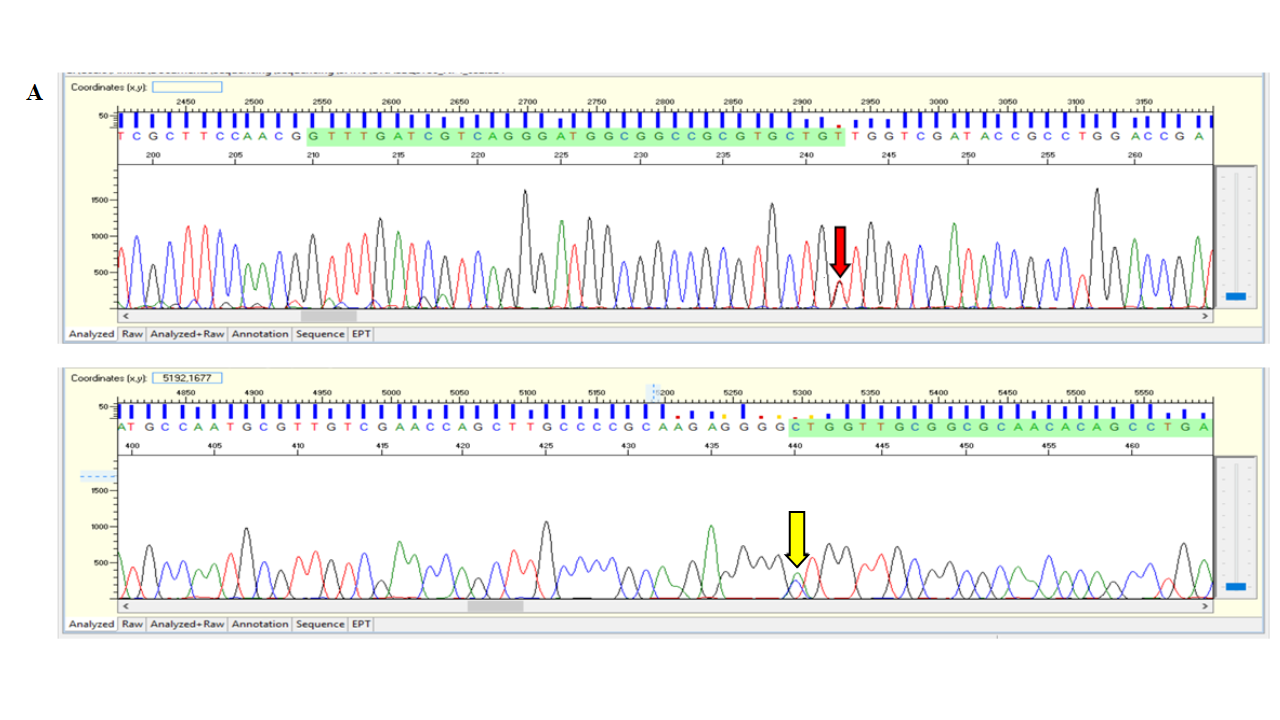
**


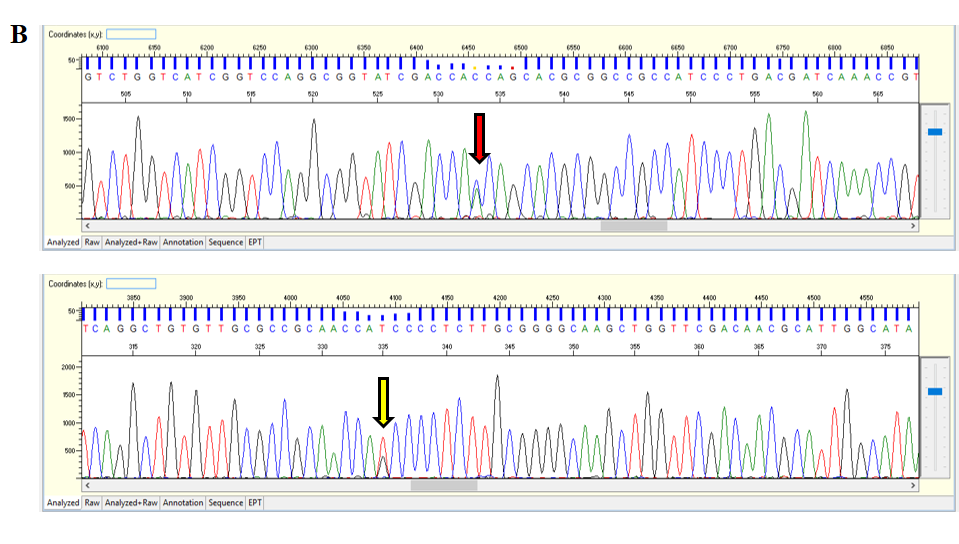


**Figure S1. Representative chromatogram of the forward (A) and reverse strands (B) of *bla*_NDM_ (EN5349).** Two sharp peaks at two nucleotide positions; G & T in 262 (red arrow) and A & C in 460 (yellow arrow). Both forward and reverse sequences were aligned to detect the mutation sequence positions (262 and 460).


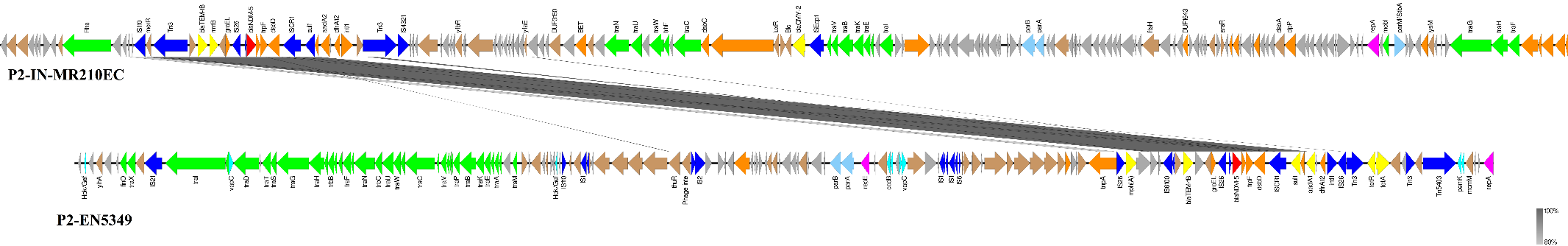


**Figure S2. Linear comparison between *bla*_NDM-5_-harbouring plasmids, P2-IN-MR210EC (IncC;~137-kb) and P2-EN5349 (IncFIA/FII; ~125-kb)**.Regions with >95% identity among plasmids are indicated in grey. Coding regions are denoted by arrows and are colored according to the gene’s functionality. Pink arrows represent genes involved in replication machinery while cyan color indicates genes for plasmid stability. *bla*_NDM-5_, other antimicrobial resistance genes and mobile element genes are indicated by red, yellow and blue arrows, respectively. Hypothetical protein-coding regions are denoted by grey arrow and conjugation module by green arrow.


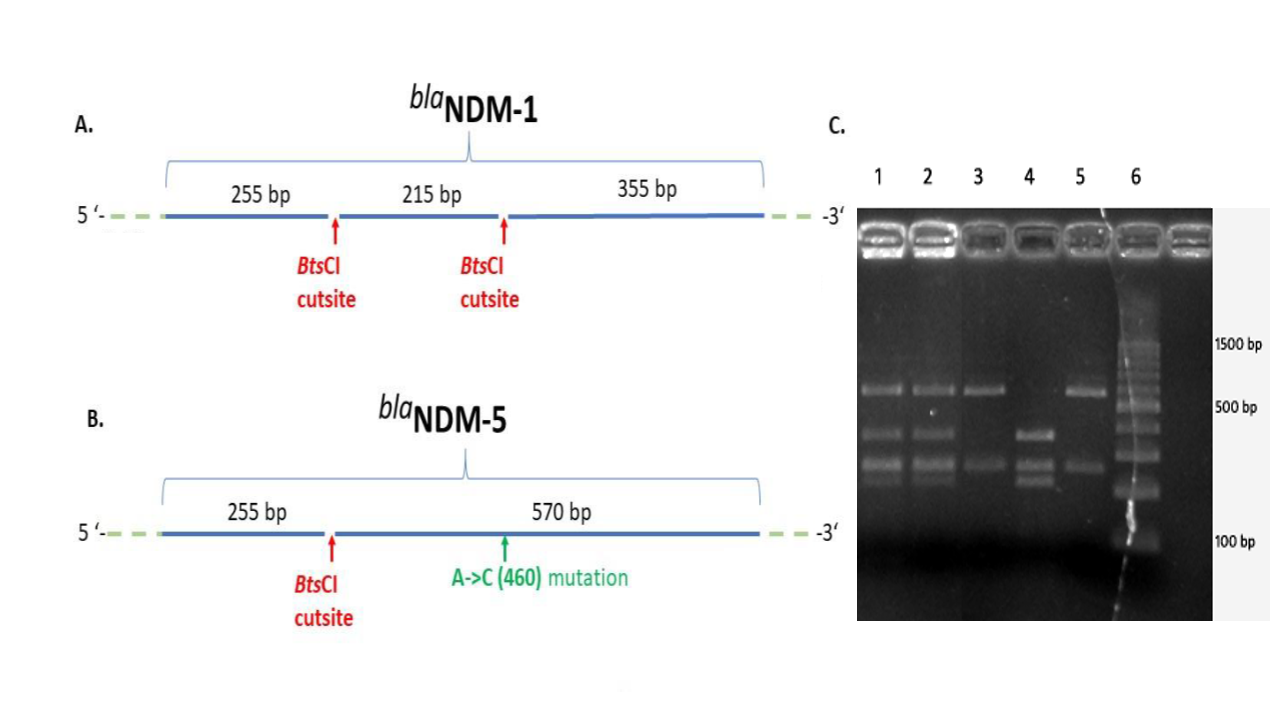


**Figure S3. Differentiation of *bla*_NDM_-variants possessing M154L mutation from *bla*_NDM-1_ by BtsCI digestion.** Diagrammatic representation of BtsCI digestion of different alleles: A. ***bla*_NDM-1_** generates three bands (215 bp, 255 bp and 355 bp); B. ***bla*_NDM-5_** generates two bands (255 bp and 570 bp) due to mutation M154L; and C. Image of agarose gel showing bands after digestion of amplified *bla*_NDM_ with BtsCI; Lane 1-2 correspond to EN5349 and IN-MR210EC generating 4 fragments such as 215 bp, 255 bp, 355 bp and 570 bp, lanes 3-5 correspond to the representative isolates possessing either *bla*_NDM-1_or*bla*_NDM-5_, Lane 6 100 bp DNA ladder.
